# Seroprevalence of *Toxoplasma gondii* in domestic pets from metropolitan regions of Panama

**DOI:** 10.1051/parasite/2017009

**Published:** 2017-03-13

**Authors:** Claudia Rengifo-Herrera, Edwin Pile, Anabel García, Alexander Pérez, Dimas Pérez, Felicia K. Nguyen, Valli de la Guardia, Rima Mcleod, Zuleima Caballero

**Affiliations:** 1 Facultad de Medicina Veterinaria, Universidad de Panamá. Campus Harmodio Árias Madrid (Curundu) Apartado 3366 Panama 4 Panama; 2 Centro de Biología Celular y Molecular de Enfermedades, Instituto de Investigaciones Científicas y Servicios de Alta Tecnología, Asociación de Interés Público (INDICASAT). Ciudad del Saber (Clayton) Apartado 0843-01103 Panama 4 Panama; 3 National Research System, National Secretary of Science, Technology and Innovation (SNI-SENACYT). Ciudad del Saber (Clayton) Apartado 0816-02852 Panama 5 Panama; 4 Fundación San Francisco de Asís (Fundasis) Apartado 0816-04057 Panama; 5 Department of Environmental and Occupational Health Sciences, University of Washington Seattle WA 98195 USA; 6 Toxoplasmosis Center, University of Chicago Medicine 5841 South Maryland ave Chicago IL 60637 USA

**Keywords:** *Toxoplasma gondii*, Seroprevalence, Dogs, Cats, Panama

## Abstract

Toxoplasmosis is a worldwide zoonotic disease but information regarding domestic animals in Central America is scarce and fragmented. The aim of this study was to determine the seroprevalence of *Toxoplasma gondii* in domestic cats and dogs in different metropolitan regions of Panama. A total of 576 samples were collected; sera from 120 cats and 456 dogs were tested using a commercial indirect enzyme-linked immunosorbent assay (ELISA). The overall seroprevalence of IgG antibodies was 30.73%. There is high seroprevalence of *T. gondii* in cats and dogs in the metropolitan regions around the Panama Canal; however, differences between these species were not significant. Statistical analysis indicated that there are relevant variables, such as the age of animals, with a direct positive relationship with seroprevalence. None of the variables related to animal welfare (veterinary attention provided, type of dwelling, and access to green areas and drinking water) were associated with seropositivity.

## Abstract (fr)

La toxoplasmose est une maladie zoonotique mondiale, mais les informations concernant les animaux domestiques d’Amérique Centrale sont rares et fragmentées. Le but de cette étude était de déterminer la séroprévalence de *Toxoplasma gondii* chez les chats et les chiens domestiques dans différentes régions métropolitaines de Panama. Au total, 576 échantillons ont été prélevés; des échantillons de sérum de 120 chats et 456 chiens ont été testés en utilisant un dosage ELISA. La séroprévalence globale des anticorps IgG était de 30.73 %. Nous avons trouvé une importante séroprévalence de *T. gondii* chez les chats et les chiens dans les régions métropolitaines autour du Canal de Panama, mais les différences entre les deux espèces n’étaient pas significatives. L’analyse statistique indique qu’il existe des variables pertinentes, comme l’âge des animaux, qui ont une relation positive avec la séroprévalence. Aucune des variables liées au bien-être des animaux (attention vétérinaire fournie, type de logement et accès aux espaces verts et à l’eau potable) n’étaient associée à la séropositivité.

## Introduction

Toxoplasmosis is one of the most common infections, with a wide geographic distribution and infects a large number of birds and mammals [11, 26, 32]. The definitive hosts are all members of the Felidae family, infected either by the ingestion of oocysts shed in the environment or tissue cysts ingested during carnivorism. Cats can excrete millions of oocysts through feces; oocysts are highly resistant in the environment, surviving for months in soil and water. After initial infection, cats acquire lifelong immunity and do not shed oocysts again after being re-infected, except if they suffer severe malnutrition or superinfection by other etiological agents such as *Isospora* sp. [11, 32].

Most seropositive cats have already shed oocysts. Because of this, there is epidemiological importance in detecting seropositive felids [4, 5, 32]. The identification of seropositive cats in a community is important to estimate past soil contamination with oocysts, determining populations with a high risk of exposure to *T. gondii* [4]. Epidemiological studies conducted worldwide have also demonstrated a significant association between positive cases in humans and contact with soil [2, 14–16, 23, 24, 33].

Dogs have also been reported to play a role in the mechanical transmission of the parasite [7–9, 20, 31, 32, 34]. Their presence in households has been considered a risk factor for infection with *T. gondii* in humans. They may contribute to transmission by spreading oocysts in the environment via shedding after ingestion of oocysts without the replication of the parasite in the intestine, which only occurs in cats. They can physically disperse oocysts due to coprophagy and rolling habits in cat excrement, contaminating their fur [8].

Currently, there is very limited data regarding the seroprevalence of *T. gondii* in domestic animals in Central America. In Panama, previous studies have shown a high seroprevalence in swine (32.1%) and cats (45.6%), indicating high levels of exposure to the parasite and a high risk of transmission to humans [3, 9].

The aim of this study was to determine the seroprevalence of *T. gondii* in domestic cats and dogs in different metropolitan regions of Panama, near the Panama Canal. In addition, variables related to demographic and animal welfare data were evaluated, to elucidate variables associated with *T. gondii* infection.

## Materials and methods

### Studied communities

This study was conducted in four regions of the metropolitan area of Panama, three of them located in Panama City, defined as: Central Region (80°17′ ~ 79°33′ W, 8°27′ ~ 8°57′ N), East Region (79°25′ ~ 79°6′ W, 9°7′ ~ 9°9′ N), and San Miguelito Region; and a fourth region located in a neighboring province, West Panama, defined as West Region (79°40′ ~ 79°54′ W, 8°58′ ~ 8°32′ N).

### Sample collection

A total of 576 blood samples were collected from both domestic cats (*n* = 120) and dogs (*n* = 456) for one year (October 2015–October 2016). The sera were obtained during spay/neuter interventions (sterilization programs) performed in the communities of studied sites (Table 1). A signed consent and approval were obtained from owners, who voluntarily included their animals in the sterilization campaign.

**Table 1.** Prevalence and surveyed data for *Toxoplasma gondii* in studied regions.

Regions studied	Communities studied	No. of pets tested	No. (%) positive cases	Species	No. of animals tested	No. (%) of positive animals	No. of animals per household	Weight (kg)	Age (months)
West	Arraiján and Chame	91	20 (21.74%)	Cats	36	5 (13.88%)	4	3.44	13.64
				Dogs	55	15 (27.27%)	1	16.37	27.03
Central	Alcalde Díaz, Chilibre, Curundu, Ancon and Juan Díaz	205	53 (25.74%)	Cats	54	16 (29.62%)	1	2.92	9.33
				Dogs	151	37 (24.50%)	1	9.69	24.72
San Miguelito	Mateo Iturralde, José Domingo Espinar, Victoriano Lorenzo, and Amelia Denis de Icaza	50	13 (26.00%)	Cats	13	0	1	2	NA
				Dogs	37	13 (35.13%)	1	14	NA
East	Mañanitas, Chepo, Pacora, and Tocumen	230	91 (39.56%)*	Cats	17	9 (52.94%)	NA	2	NA
				Dogs	213	82 (38.49%)	NA	14	NA
Total		576	177 (30.73%)	Cats	120	30 (25.00%)	–	–	–
				Dogs	456	147 (32.23%)			

* (*p* < 0.05);

NA = information not collected through surveys.

Samples were collected by puncturing either the cephalic, saphenous or jugular vein and 1–3 mL of blood was extracted and placed into tubes for serology, kept in an ice cooler and transported to the laboratory. Serology tubes were centrifuged at 3000 rpm for 10 min, and the sera stored at −20 °C until analysis.

### Serologic examination

An indirect enzyme-linked immunosorbent assay (ELISA) was used for detection of IgG antibodies against *Toxoplasma gondii* in sera (Multi-species ID Screen^®^ Toxoplasmosis Indirect*,* IDVET, Montpellier, France), according to the manufacturer’s instructions. The cut-off for positive results was defined with an optical density of 0.350 (OD > 0.350). Absorbance was measured at 450 nm with an automatic 96-well plate reader (BioTek Synergy HT, VT, USA).

### Demographic and animal welfare data

A questionnaire was provided to pet owners in the Central, West, and San Miguelito Regions, to identify the characteristics of the species (sex, age, weight, species, and number of animals per household) and level of animal welfare (veterinary attention provided, type of dwelling, access to green areas, drinking water, and disease information).

### Statistical analysis

The sample size was verified by clearing the margin of error, using the equation described by Schwartz [30]. Data were tabulated and evaluated using exploratory analysis with the aid of a statistical computer program [27]. The dimensionality of the data set was reduced using a principal component analysis (PCA) [18, 19].

To understand the drivers of the variables in the surveyed population, we conducted exploratory analysis techniques by partitioning the data set using a grouping method in which each observation belongs to the group whose mean value is closest [22]. Cross-validation was established to reduce the margin of error. All data were described through measures of central tendency and minimum and maximum values.

## Results

Across all studied regions of Panama’s metropolitan area, mean seroprevalence for *T. gondii* in the sampled domestic pets was 30.73% (margin of error = 0.04%). Prevalence results were consistent for all dogs (32.23%) and cats (25.00%) included in the study, but the difference was not significant (chi-square test, *p* > 0.05).

The percentage of positive dogs was relatively high in all the studied regions (24.50%–38.49%). For cats, this percentage varied among regions, from none in the San Miguelito Region to a high number of positive animals in the East Region. Although the number of collected samples was higher for dogs (*n* = 456) than cats (*n* = 120), the differences in prevalence between regions and for both species by region were not significant (chi-square test, *p* > 0.05). On the other hand, the East Region showed a higher prevalence of *T. gondii* for pets (39.56%) compared to the other studied regions (ANOVA; *p* < 0.05).

The survey defined the number of animals per household in all the studied regions (except the East Region), indicating that there was at least one animal per surveyed household, except for the West Region where the number of animals was higher, with some households reporting up to four cats (Table 1).

**Figure 1.**
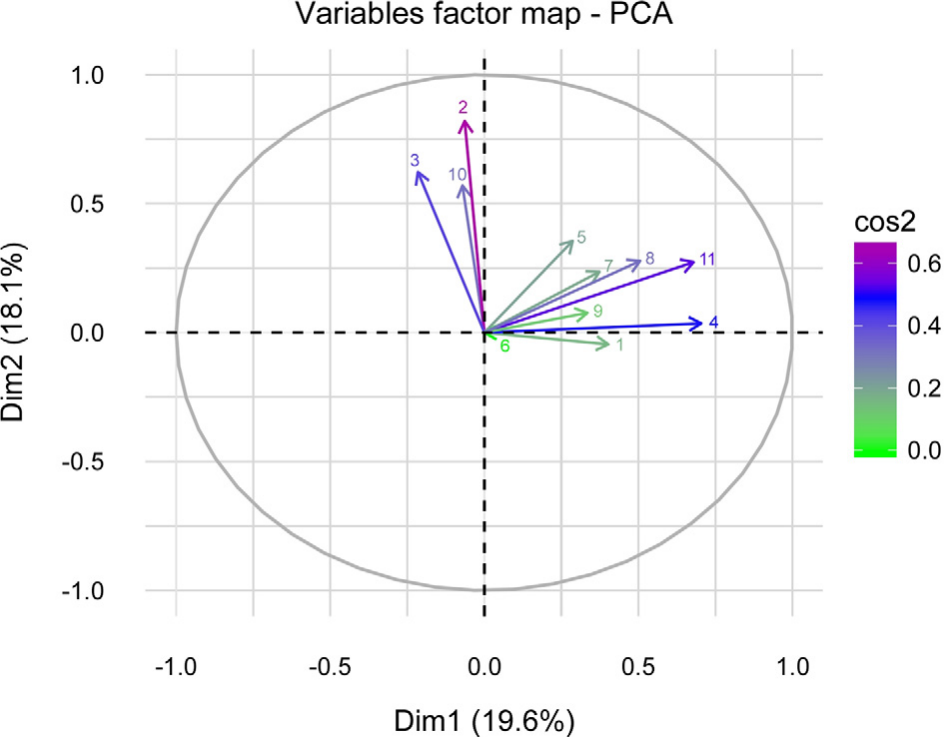
Principal component analysis of variables (PCA). The factor map helps to visualize the cluster of correlated variables in groups (≅90°). Cos2 is the gradient of quality to highlight the most important variables in explaining the variations retained by the principal components. Dimension 1 and 2 (Dim1 and 2) is the space where variables are expressed (<38% of variance). The distribution of the surveyed population through variables is also observed. Variables analyzed: (1) sex, (2) weight, (3) age, (4) number of animal per household, (5) veterinary attention, (6) type of dwelling, (7) access to green areas, (8) drinking water, (9) disease information, (10) prevalence and (11) region.

To evaluate the behavior of the variables collected through the survey in explaining their influence on *T. gondii* prevalence, a principal component analysis (PCA) was conducted using a factor map (Fig. 1). The PCA revealed those variables with higher quality in the study, although these were only able to explain 38% of the variance. The PCA is dominated by two main groups of highly correlated variables. First, the variables age, weight, and prevalence showed a positive correlation and the highest quality. Second, region, number of animals per household, and those variables related to the level of animal welfare (i.e. veterinary attention, type of dwelling, access to green areas, drinking water, and disease information) also showed a positive correlation. Of all these variables, region and number of animals per household showed the highest quality. On the other hand, these variables showed homogeneous behavior among the West, Central, and San Miguelito Regions.

## Discussion

Prevalence studies in dogs and cats have been conducted around the world reporting a wide range of seroprevalence for *T. gondii.* In Bogota (Colombia), Dubey et al. reported seroprevalence of 16.8% in unwanted dogs [6]. In Brazil, Brandao et al. [1] and Rodrigues et al. [29] reported 40.90% and 43.1% seroprevalence in domestic dogs from Minas Gerais and Mato Grosso (Pantanal), respectively. In Madrid (Spain), a seroprevalence of 32.3% was found in stray cats, whereas in Thailand and New Caledonia, seroprevalence values were 11.0% and 32.8%, respectively. Likewise, in the last two regions, seroprevalence in dogs was 9.4% and 50%, correspondingly [17, 21, 28]. In Panama City, reports on seroprevalence in domestic pets are limited. In this study, seroprevalence values for dogs and cats in the studied regions were 32.23% and 25.00%, respectively, with a mean prevalence of 30.73% (*p* > 0.05). These results are similar to those reported for Panama City, where the seroprevalence in domestic cats was 45.6%, suggesting that dogs are implicated in the dynamics of the disease [8]. However, little is known about the factors involved in the epidemiology of *Toxoplasma* infections in domestic pets in Panama, especially in the metropolitan region.

This study sampled four regions surrounding the metropolitan area of Panama City, where no significant difference was found in prevalence between dogs and cats. This reveals that the infection rate is similar, suggesting that both species have the same probability of being infected with the parasite.

Socioeconomic and environmental factors have been associated with transmission and consequently, a higher prevalence for *T. gondii*. For example, intake of raw or undercooked meat containing tissue cysts, contact with oocysts-contaminated food, water, and soil, and mother to offspring transmission, are some factors involved in the epidemiology of this disease [29]. For dogs, coprophagy and rolling in cat excrement are some of the risk factors involved in transmission [8]. In the regions studied, aspects such as the number of animals (including different species) interacting with dogs and cats, animal habits and pet care (e.g. bathing and grooming) might increase exposure to the parasite, influencing the high infection rate (P = 21.74%–39.56%).

The East Region showed the highest prevalence (39.56%) with statistical significance among the studied regions (ANOVA, *p* < 0.05). Unfortunately, no surveys were conducted; therefore, we are limited in explaining these results. However, socioeconomic, cultural, and demographic components, as well as the type and location of domestic animal species present, could be relevant to understand the dynamics of the animal population, and consequently the disease [13]. In suburban areas, a statistical trend is observed showing an increasing number of canines closely related with high poverty [25]. In the East Region of Panama City, the human density is low (<500−2000 inhabitants per km^2^) and the region has the lowest socioeconomic status. Therefore, the environment is conducive to a larger number of animals coexisting with humans, with perhaps a greater probability of contact with the parasite and a greater number of infected individuals [12].

The principal component analysis (PCA) showed homogeneous behavior according to the surveyed variables analyzed for West, Central, and San Miguelito Regions, forming a single group population possibly due to their similar demographic characteristics. These three regions have the highest human density in the country (>500 inhabitants per km^2^) and a socioeconomic middle class status [12]. Therefore, it is possible that these characteristics provide a similar environment for domestic pets, defining the prevalence of the disease (P = 21.74%–26.00%).

The PCA was used to correct the dimension of the collected data (error range = 0.04%). Compiled information in surveys includes variables related to the characteristics of the species and the level of animal welfare. The correlation coefficient indicated an interdependency of variables and a low coefficient (38%), suggesting more variables should be included in order to increase the variance.

The most important variables identified with this analysis were weight, age, prevalence, region, and number of animals per household. The correlations suggest that age and weight have an influence on the prevalence of the disease (Fig. 1). Older animals tend to reach a greater weight than younger animals, influencing the increment of prevalence in the population, mainly because they have more opportunities to have contact with the parasite over a longer time. These results were consistent with those of a study conducted in Ethiopia, where seropositivity was related to age [10].

This study was an attempt to identify those factors involved in the dynamics of *Toxoplasma* infection in domestic pets in Panama. Since cats and dogs are the most popular pet animals, their infections likely affect their owners and others living in their environment. Such information is critical to establishing prevention strategies to minimize this neglected disease.

## Conclusion

The seroprevalence of 25% and 32.23% for *T. gondii* serum antibodies in cats and dogs in different communities of the metropolitan regions of Panama indicates that pet animals are likely to represent a risk factor for the transmission of *T. gondii* in humans, although socioeconomic and environmental factors may also play an important role in transmission of the disease.

## Conflict of interest

The authors declare that they have no conflict of interest.
